# A Digital Therapeutic Intervention Delivering Biofeedback for Panic Attacks (PanicMechanic): Feasibility and Usability Study

**DOI:** 10.2196/32982

**Published:** 2022-02-03

**Authors:** Ellen McGinnis, Aisling O'Leary, Reed Gurchiek, William E Copeland, Ryan McGinnis

**Affiliations:** 1 Department of Psychiatry University of Vermont Medical Center Burlington, VT United States; 2 M-Sense Research Group University of Vermont Burlington, VT United States

**Keywords:** mental health, mHealth, biofeedback, panic attack, digital medicine, app, mobile health, application, biofeedback, mobile phone

## Abstract

**Background:**

Panic attacks (PAs) are an impairing mental health problem that affects >11% of adults every year. PAs are episodic, and it is difficult to predict when or where they may occur; thus, they are challenging to study and treat.

**Objective:**

The aim of this study is to present PanicMechanic, a novel mobile health app that captures heart rate–based data and delivers biofeedback during PAs.

**Methods:**

In our first analysis, we leveraged this tool to capture profiles of real-world PAs in the largest sample to date (148 attacks from 50 users). In our second analysis, we present the results from a pilot study to assess the usefulness of PanicMechanic as a PA intervention (N=18).

**Results:**

The results demonstrate that heart rate fluctuates by about 15 beats per minute during a PA and takes approximately 30 seconds to return to baseline from peak, cycling approximately 4 times during each attack despite the consistently decreasing anxiety ratings. Thoughts about health were the most common trigger and potential lifestyle contributors include slightly worse stress, sleep, and eating habits and slightly less exercise and drug or alcohol consumption than typical.

**Conclusions:**

The pilot study revealed that PanicMechanic is largely feasible to use but would be made more so with modifications to the app and the integration of consumer wearables. Similarly, participants found PanicMechanic useful, with 94% (15/16) indicating that they would recommend PanicMechanic to others who have PAs. These results highlight the need for future development and a controlled trial to establish the effectiveness of this digital therapeutic for preventing PAs.

## Introduction

### Background

Almost one-third (>28%) of adults have experienced a panic attack (PA) and >11% have experienced one in the past 12 months [1]. People experiencing PAs exhibit impairment in physical and emotional health as well as in occupational and financial functioning. They also have increased use of health care facilities, emergency departments, and psychoactive drugs [2]. A PA is defined as “an abrupt surge of intense fear or discomfort that reaches a peak within minutes,” characterized by rapid activation of stress-related physiology and fear-related cognitions [3]. Although the body’s ability for rapid physiological activation is evolutionarily advantageous (eg, to provide a bodily response to a direct predatory attack [4]), the generation of this response during PAs, which are inherently *unexpected,* impairs daily functioning [3].

### Quantifying PAs

Given their critical evolutionary role in the body’s acute response to stress [5] and measurement feasibility, heart rate (HR) and respiration variables have been studied as key metrics in PA research. For instance, HR was observed to increase significantly 1 minute before patient-identified PA onset and remain at a level significantly higher than the resting state for the approximately a 10-minute duration of the PA [6]. However, other studies measuring HR during real-world PAs demonstrated mixed results such that only a moderate percentage of examined PAs (37%-68%) were found to exhibit rapid HR increases compared with non-PA periods. Similarly, respiration changes have been observed before PA and during the PA as characterized by changes in tidal volume and end-tidal PCO_2_, but these PA-related values did not differ significantly from typical resting states [7-9].

As PAs are unexpected and episodic, they are difficult to capture in real-world situations. To collect and present sufficient data, studies on PA physiology have (1) presented spontaneous PA data as case studies [10,11], (2) induced PAs medically in the laboratory [12], or (3) focused on a subsample of 4% of people having PAs who have panic disorder (PD) [6,13], which is characterized by recurrent PAs with persistent worry between PAs. There is evidence to suggest that PA physiology differs in severity depending on how the PA is induced [12] and participant symptomatology [6]; thus, generalizations to real-world PAs for individuals without PD are limited. The sample size across studies is also relatively low. In a study of individuals with PD, 43 participants were monitored for 6 continuous days and 13 unexpected PAs were observed, demonstrating that high effort and participation is necessary to collect data on even a small number of episodic PAs. In the 3 studies that aimed to capture the average physiological changes during real-world PAs in individuals without PD, the combined number of PAs was only 50 [7-9] including several PAs within individuals. Thus, there is a clear need for additional studies of PA physiology, particularly in individuals without PD.

A key factor that has likely limited previous studies of PA physiology is the availability of technologies for providing noninvasive measurements in real-world environments. For instance, one of the HR studies described above conducted continuous ambulatory monitoring with 9 wearable sensors connected by wires to a data monitor worn in a fanny pack [6]. This approach imposes a significant burden on researchers and participants alike. Less cumbersome equipment does exist [14-16], yet these specialized devices can be expensive and burdensome to carry around, limiting accessibility. To better capture a representative picture of real-world PAs, more feasible and accessible tools are needed to measure PAs wherever and whenever they occur.

### Treating PAs

Although there is limited evidence demonstrating the real-time physiological response to PAs, there is more research on their treatment. It is critical that individuals experiencing PAs have access to feasible and evidence-based interventions as they tend to seek help at significantly higher rates (46%) than individuals with any other axis 1 disorder each year [2,17]. Despite increased treatment-seeking, only 18% of services delivered to these individuals are based on evidence specific to PA reduction [2], as most treatments are intended for general mental health impairment (ie, anxiety). Existing PA treatments can be categorized by their mechanism of action as follows: (1) actively attempting to prevent physiological symptoms (avoidance techniques) or (2) reframing the patient’s perspective of their physiological symptoms (approach techniques).

Avoidance techniques, such as meditation or progressive muscle relaxation, focus on preventing physiological symptoms at onset. In individuals with general anxiety, avoidance techniques have been found to induce (not reduce) anxiety in a significant number of patients (17%-54%) [18]. For PAs specifically, prescribed relaxation demonstrates poorer effectiveness and significantly higher attrition than other treatment options [19]. Psychopharmacological intervention (eg, benzodiazepines) is another potential avoidance technique for treating PAs. Although this approach effectively stops the physiological symptoms of PAs, it does not decrease the likelihood of future PAs and has serious clinical side effects (eg, dependence, rebound anxiety, and memory impairment) that reduce quality of life [20]. Thus, there is little evidence demonstrating that avoidance techniques are effective in addressing the underlying mechanisms that drive PAs and some of these approaches have significant side effects.

Approach techniques aim to help patients think about their physiological symptoms differently and confront them. Psychotherapies such as cognitive behavioral therapy with interoceptive exposure involve reframing panic-related thoughts and learning to experience symptoms with less panic via in vivo exposure (eg, by inducing PA symptoms, such as dizziness, in a session by spinning the patient in a chair). These therapies are very effective in reducing PA frequency and severity [19] but require a weekly 1-hour visit with a trained and licensed clinician for 12 consecutive weeks. Unfortunately, access to these evidence-based therapies is limited [1], which motivates the development of alternative approaches for treating PAs that do not require the presence of a licensed mental health professional.

Biofeedback is an approach technique in which some form of involuntary physiology (eg, respiratory rate, electroencephalogram, or HR) is continuously measured over time and simultaneously displayed back to the user. During biofeedback, the user is trained to improve their health by learning to regulate internal bodily processes that typically occur involuntarily [21]. Biofeedback with breathing training (BT) is one of the most commonly studied biofeedback techniques for treating PAs. This approach, which requires a specialized device (ie, capnometer) to measure breathing data and display it back to the patient in real time, helps patients learn how to raise or lower their end-tidal PCO_2_. In 2 studies of patients with PD, a 4-week biofeedback with BT intervention (two 17-minute sessions per day) was shown to be effective at reducing PA frequency and severity [15,22] at 1 year follow-up. However, studies have shown that individuals with PD exhibit breathing irregularities even when not experiencing a PA [23] and thus the effectiveness of this treatment may be limited to those with PD [24]. Other forms of biofeedback without BT have been shown to be effective for treating more general anxiety [25,26] and thus could also be effective for treating PAs in those without PD. For example, a meta-analysis of 24 studies investigating the impact of HR variability biofeedback on stress and anxiety revealed a rather large effect (Hedges *g*=0.83) [26]. Although these data suggest that biofeedback can improve anxiety-related symptoms across physiology, treatment effectiveness specific to PAs in persons without PD remains unknown. Biofeedback is thought to provide effective intervention for PAs because it allows users to “feel more in control of their bodily reactions and react less fearfully to them,” thus ending the cycle of panic by acquiring a sense of mastery [17]. In support of this theory, *perceived control* was found to be associated with the effectiveness of both biofeedback with BT and cognitive behavioral therapy [27]. HR, but not respiration variables, has been significantly related to patient-reported rating of *fear of losing control* [6], indicating that HR may be an ideal choice for such interventional strategies. HR is significantly higher during PAs [6] than in a resting state for those who have PA, but there is no evidence to suggest that individuals with PAs (but not PD) experience differences in HR during typical resting states. Approach techniques that demonstrate long-term reduction in panic and anxiety focus on confronting symptoms when they are active, even attempting to reproduce similar symptoms while in a therapist’s office (eg, interoceptive therapy). Now that we have the technology to provide the tools and guidance during an episodic PA, biofeedback could be especially effective during a PA, when an individual can immediately observe their HR fluctuations; however, this has not yet been examined in PAs.

**Figure 1:**
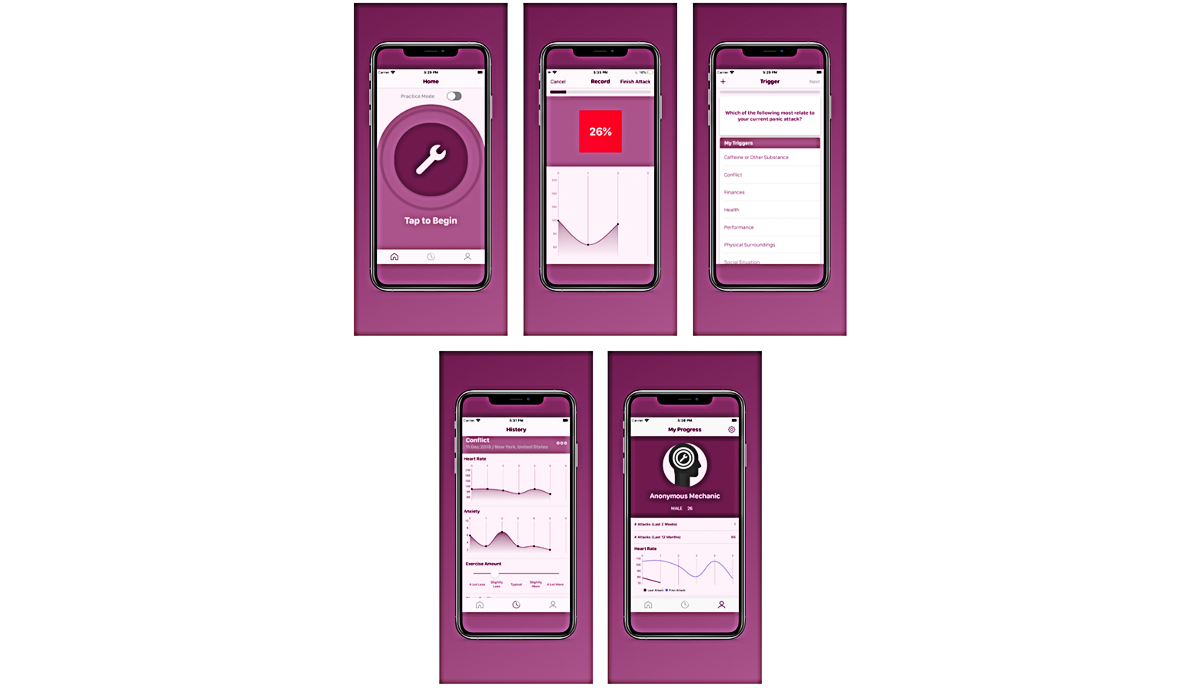
PanicMechanic mobile health app screens. The PanicMechanic mHealth app is available wherever and whenever a user experiences a PA (screen 1). It provides biofeedback through objective measurement of HR during the PA (screen 2) and allows users to capture their perceived anxiety throughout the PA and identify their behavioral and thought triggers (screens 3 and 4). These data are aggregated over time to allow users to track their progress and identify trends that may be helpful for preventing future PAs (screens 4 and 5).

To address the unmet needs of (1) quantifying PAs in individuals in more representative and larger samples and with less burden and (2) offering an accessible, biofeedback-based treatment option for PAs, our research team has developed a digital therapeutic called PanicMechanic (Figure 1). This mobile health (mHealth) app can accurately and feasibly collect HR data during a PA using only a smartphone and use these data to provide HR-based biofeedback to users during their PAs [28]. This tool can profile remote PAs, which was limited by technology so far, but the usefulness of this novel intervention still needs to be assessed to inform future efficacy trials. Thus, in this study, we aim to present our novel digital therapeutic PanicMechanic and leverage it as a feasible and scalable data collection tool to capture profiles of real-world PAs in a sample of help-seeking PanicMechanic users (analysis 1) and conduct a pilot study to assess the feasibility and usefulness of PanicMechanic as a PA intervention in a convenience sample of university students (analysis 2).

## Methods

To address these aims, we first introduce PanicMechanic and then describe the data collection and analysis procedures used to capture the profiles of real-world PAs. Finally, we describe the data collection and analysis procedures used to pilot PanicMechanic as a PA intervention.

### PanicMechanic

**Figure 2:**
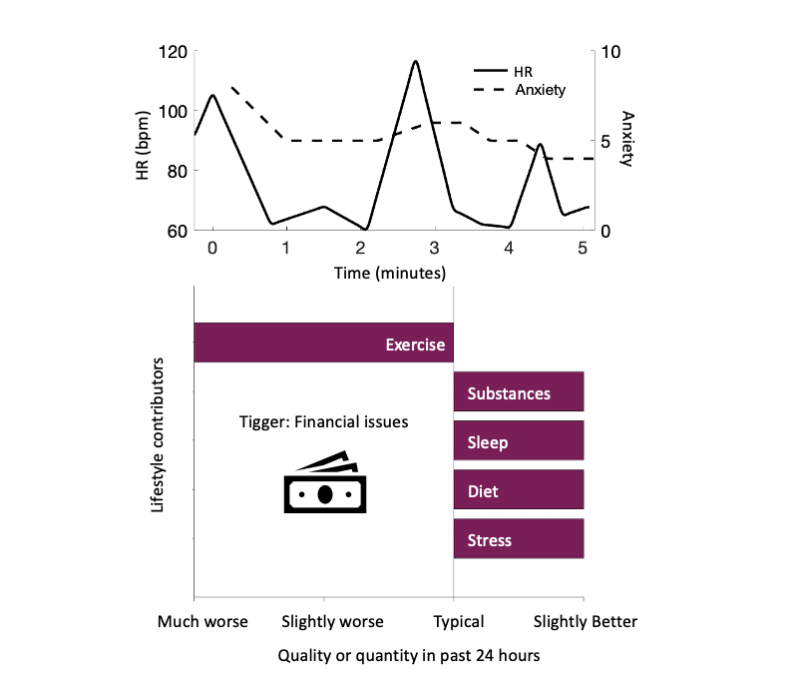
Example of panic attack case recorded with PanicMechanic. bpm: beats per minute; HR: heart rate. Data from a PA tracked by a PanicMechanic user are reported here. The app tracked HR (bpm) and anxiety ratings (on a scale of 0 to 10; low to high) during attacks (top). This attack lasted for approximately 5 minutes, during which time HR showed 3 distinct peaks and anxiety ratings generally decreased. The app also allowed users to identify lifestyle factors and triggers that may have contributed to the attack (bottom). Data were tracked over time and reported back to the user (Figure 1) to help them identify trends that may be helpful for preventing future PAs.

PanicMechanic is a commercially available mHealth app (available on Android and iPhone operating system) developed by our team and released in April 2020 that guides users through their PAs (Figure 1). The app provides biofeedback through objective measurements of HR during the PA, which are enabled via an analog to reflective photoplethysmography provided by the smartphone’s camera and validated algorithms [29]. For photoplethysmography, the camera is used to assess changes in the color of the fingertip associated with blood being pumped by the heart through the capillary bed of the fingertip. The app also allows users to rate their anxiety levels throughout the PA and choose from a selection of and record information about lifestyle contributors and triggers (see Figure 2 for data from an example user’s PA). App features were developed by a clinical psychologist with experience in treating PAs, and leveraged best practices in clinical psychology while considering input from a variety of individuals who undergo or who have undergone PAs. Our previous work has demonstrated the feasibility of this measurement modality during PAs [29,30]. Upon download, users complete a brief tutorial and are encouraged to open the app whenever they start to experience a PA. During a PA, users are first instructed to place an index finger on the lens of their phone camera with the flash activated to record their HR for the first time. The app cycles through screens that display a graph of HR over time (biofeedback) and prompts the users to make additional HR measurements, rate their anxiety level, and answer questions about lifestyle contributors (exercise, sleep, nutrition, stress, and substance use) and triggers for their PA. The screens provide encouraging messages, such as “You got this!” throughout the attack and using the data from previously logged attacks, the app also provides an estimate of the time that remains in the user’s attack. PanicMechanic aggregates user data over time to allow identification of trends (eg, HR, anxiety ratings, and most common triggers) that may help users prevent future attacks.

### Capturing Profiles of Real-world PAs (Analysis 1)

To capture profiles of real-world PAs, we considered data from PanicMechanic users in the 1-year period from April 2020 to April 2021. All the users of the app agreed to its terms and conditions, which included the statement, “Your User Content might be anonymized and used for research purposes.” As of April 2021, PA data were available from 148 anonymized PanicMechanic users.

Data from the first PA tracked by these users were used to examine the average profile of HR and anxiety ratings throughout an attack. The inclusion criteria for considering the first PA from a given user were that the HR time series had to have at least 4 HR samples (80/148, 54.1% rejected) and a clear peak (18/148, 12.2% rejected). These criteria led us to reject, for example, instances when app use was terminated before the end of the PA (before a peak HR occurred) or commenced after the peak HR had occurred. These data were leveraged to compute the ensemble average time series of HR and subjective anxiety ratings to capture the physiological profile of real-world PAs and explore their relationships during the PA.

We cannot control when users begin using the app during their PA; hence, to compute the ensemble time series, it was necessary to express the time series relative to a standardized instant in time. Thus, all time stamps were expressed relative to the first peak in the HR signal as this was an easily identifiable physiological feature, was expected to characterize a typical PA [6], and was common across many of the tracked PAs. As the time stamps did not exactly match across the time series, it was necessary to linearly interpolate the HR data over a uniform grid (0.01-second intervals) for the time interval (−15 and 60 seconds, ie, only data 15 seconds before and 60 seconds after the first HR peak were considered) before averaging. Time series were then smoothed using a low-pass filter (zero-phase, fourth order, Butterworth filter, and 0.1 Hz cutoff frequency) and averaged. If a time series contained data only for time stamps within (but not the entire) a time interval, it was included in the ensemble average only for those time stamps for which data were recorded or could be interpolated. For example, if a time series had data at time stamps −17, −5, 0, 20, and 33 seconds, it would contribute to the ensemble average only for the subinterval (−15 and 33 seconds). Ensemble average time series of subjective anxiety rating data (n=48) were processed in the same way as the HR data (n=50) for PAs that met the inclusion criteria.

On capturing the profiles of real-world first recorded PAs, we also extracted information from each PA about the typical duration of recordings, severity (in terms of peak HR and anxiety rating), and information about lifestyle contributors and triggers. To provide a more comprehensive picture of these factors, we considered all the data from the first 4 PA recordings that met the inclusion criteria from 148 PanicMechanic users.

### PanicMechanic as a PA Intervention (Analysis 2)

To assess the feasibility and usefulness of PanicMechanic as a PA intervention, we considered data from a pilot study of PanicMechanic use among university students. This demographic was chosen as a convenience sample because the majority of university students have smartphones and are open to using mental health apps [31] and because PAs have been shown to rise significantly during the transition to adulthood [32]. Email, social media, and flyer advertisements were used to recruit 18 participants at a northeastern public university in March 2020, April 2020, and May 2020, notably (and coincidently) just after the COVID-19 pandemic–related stay-at-home order was issued in the northeastern United States. Interested persons were screened via phone for eligibility with the following inclusion criteria: must own an Apple iPhone (Android version was not bug-free at the time of the study), be at least 18 years old, report experiencing a PA in the past month, and have university student status.

The participants provided written informed consent before completing a baseline assessment survey. They were given access to PanicMechanic on their personal smartphones and viewed the app’s tutorial. They were instructed to use the app whenever they experienced a PA during the following 12 weeks. Throughout the study period, the participants completed 2-minute weekly web-based surveys about their PA and were administered a 15-minute web-based follow-up survey about their experience with using the app 1 week after the study period. Upon study completion, participants were compensated with Amazon gift cards worth up to US $50. All data collection activities were approved by the institutional review board of the University of Vermont (CHRBSS 00000747).

The baseline assessment survey included 42 items that captured participant demographics; PA symptom assessment using the Structured Clinical Interview for Diagnostic and Statistical Manual for Mental Disorders, which is a standardized semistructured interview; and previous treatment and mental health history. During the 2-minute weekly survey, if the participant self-reported capturing a PA with PanicMechanic, they also rated how difficult it was to use the app and indicated the ways (if any) in which the app was helpful. The follow-up survey included open-ended questions about helpfulness and challenges of app use, an indication of whether the participant would use the app in the future, and if they would recommend the app to others who experience PAs.

Descriptive data for participants in the pilot study were available, including demographic characteristics (N=18) and their PanicMechanic use (n=16; of the 18 participants, 2 [11%] participants did not participate beyond the first week of the study period and were lost to follow-up). Content analysis was conducted on qualitative data responses to 2 open-ended items in the follow-up survey at the conclusion of the 12-week intervention: “Overall, in what ways did you find app use helpful” and “Overall, what challenges did you face in using the app during panic attacks?” Content analysis followed the steps detailed in a previous work [33], including first inductive and then deductive analyses to understand the data. First, 2 raters independently conducted inductive content analysis that involved reading all the participant responses, making notes on their content, grouping them, and organizing the responses into categories [33]. Next, the raters jointly conducted deductive content analysis that involved developing a structured matrix of categories, coding the data into those categories, and discussing how those data compare to expectations [33]. Any discrepancies between the 2 raters were blindly analyzed by a third rater and majority ruled. Consensus categories, descriptions, percentages of total responses, and example responses are presented.

## Results

### Profiles of Real-world PAs (Analysis 1)

A total of 148 PanicMechanic users tracked at least some HR data from at least one PA. In accordance with the iOS app privacy policy, no demographic data were required to download and use the app; thus, we were not able to report any demographic data besides the knowledge that all 148 users had unique and verified email addresses. PanicMechanic had been featured on news media outlets in April 2020 when it became available but had no paid advertising to promote it. Figure 2 provides an example of the data recorded by PanicMechanic during a PA tracked by a user, including the time series of HR and anxiety ratings (top), lifestyle contributors, and the PA trigger (bottom). This example PA lasted just over 5 minutes, during which time you could see a decreasing trend in anxiety rating and 3 clear instances when the user’s HR peaked (at between 90 and 115 beats per minute [bpm]) and then returned to baseline (at between 60 and 70 bpm). Before this attack, triggered by financial issues, the user reported much worse exercise but slightly better diet, sleep, substance use, and stress than typical. It should be noted that significant heterogeneity was observed in HR patterns across PanicMechanic users, including in the timing, amplitude, and duration of HR fluctuations and particularly following the relatively consistent pattern of initial HR peak and return to baseline.

**Figure 3:**
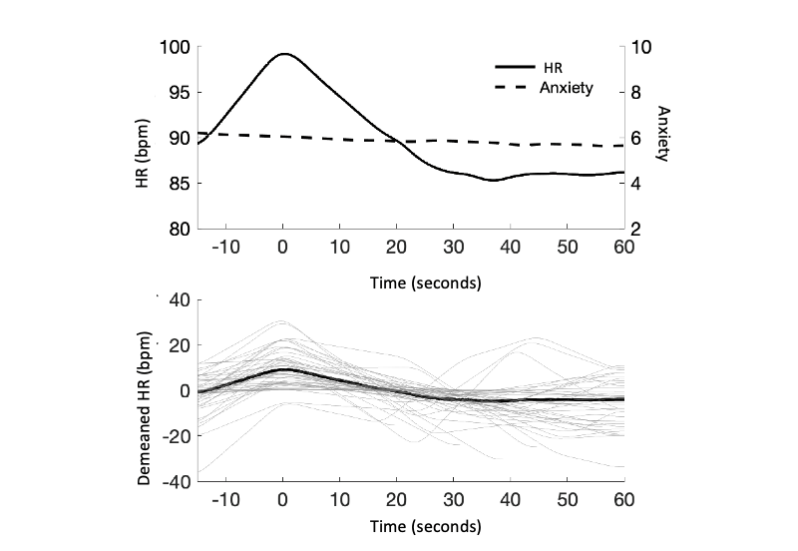
Mean heart rate (HR) and anxiety level during panic attacks. bpm: beats per minute. This figure shows ensemble average HR (solid, left axis, N=50) and subjective anxiety rating (dashed, right axis, N=48) responses for the first PA measured by PanicMechanic users (top). Both time series are expressed relative to the first peak in the HR signal. Demeaned HR recordings (gray, bottom) used for computing the ensemble average (black, bottom) demonstrate the variety in HR trajectories observed.

**Figure 4:**
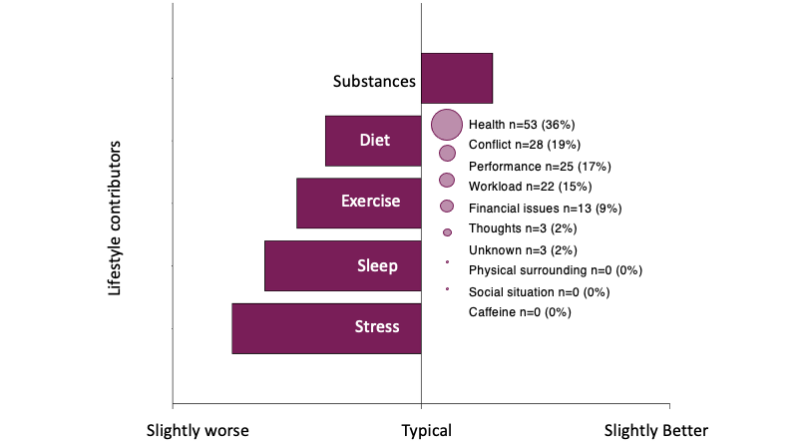
Frequencies of panic attack data. This figure shows participant-reported PA data including mean scores for potential lifestyle contributors that may impact the likelihood of experiencing a PA and the frequency of triggers identified as being responsible for inducing the PA. Lifestyle contributor responses were on a 5-point scale from *a lot worse or less* to *a lot better or more* compared with *typical*. Stress, diet, sleep quality, and exercise were all reported as slightly worse, whereas substance use was reported as slightly better immediately before the PA. Thoughts about health were the most common trigger followed by conflict, performance, and workload.

Figure 3 (top) shows the mean HR for the first PA recorded by 50 users with at least 4 HR measurements demonstrating activation (an increase to peak) and recovery (a decrease from peak) slopes. The mean peak HR was approximately 98 (SD 21.56) bpm. It appeared to take approximately 30 seconds from peak HR for a significant recovery down to 85 bpm, which was maintained for an average of 30 seconds. Subjective anxiety rating per minute (range 0-10) maintained a weak yet significant recovery slope (E=−0.43, SE 0.001; *P*<.001) from peak HR to PA end, which appears to be insensitive to specific changes in HR. Figure 3 (bottom) also demonstrates individual heterogeneity across the demeaned 50 recorded first PAs. Several individual PAs appeared to show a secondary smaller peak HR, which could indicate a cyclical HR pattern for a subset of users that was also exhibited in the example PA presented in Figure 2. The average PA recording lasted 4.64 (SD 6.27) minutes. Exercise amount, stress level, sleep, and eating habits were all *slightly worse* to *typical* and substance use was *typical* to *slightly better* during the 24 hours before a PA (Figure 4). The most commonly identified triggers out of the given choices were *health*, *conflict*, *performance,* and *workload* (Figure 4).

### PanicMechanic as a PA Intervention (Analysis 2)

**Figure 5:**
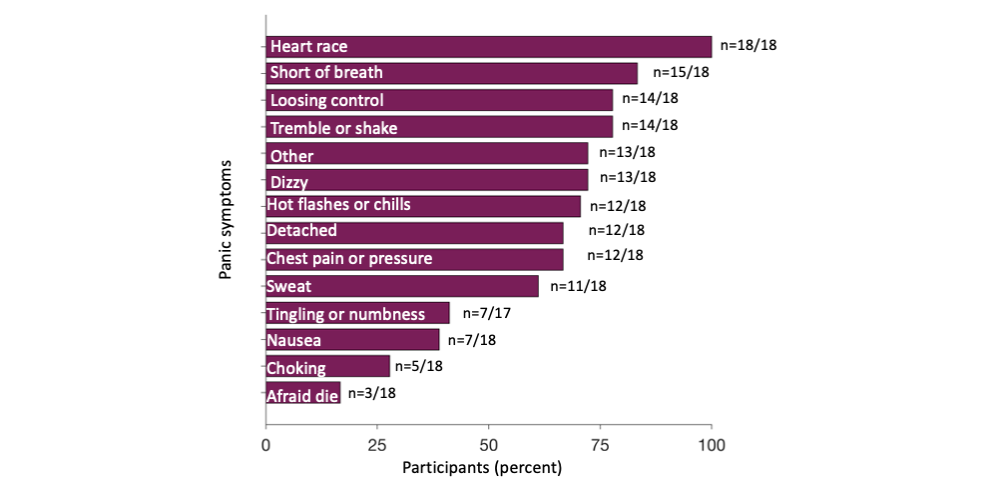
Symptoms of panic attack among the participants. This figure shows that the most common PA symptom is racing HR, which was experienced by 100% (16/16) of participants and supports our choice to consider HR biofeedback for PAs. Shortness of breath was the next most common symptom, experienced by 83% (13/16) of participants. The number of responses range from 17 to 18 owing to some missing responses.

The demographics of the 18 study participants aged 19-35 years (Table 1) indicate that most of them identified as women, were White, had diagnosed mental health disorder, and had previously sought services for their panic. None of the participants had received biofeedback previously. Participants cited an increase in PA frequency, severity, and duration (mean 5.94, SD 2.38 on a scale of 1-10; 1=not at all increased, 5=somewhat increased, 10=extremely increased) owing to the COVID-19 pandemic, which began shortly before the study recruitment commenced. The most commonly reported panic symptom was a racing, pounding, or skipping heart (16/16, 100%) followed by shortness of breath (13/16, 83%), trembling or shaking (12/16, 78%), and feeling crazy or a loss of control (12/16, 78%; Figure 5). Over the 3-month intervention, the following information was collected in weekly surveys: 94% (15/16) of the participants self-reported to have experienced 123 attacks, of which 39 (31.7%) were self-reported as recorded in the app. The reasons for missing PA recordings are listed in Table 2. Of the 10 participants, 9 (90%) participants with a valid recorded attack (recorded at least four HR data points, which would take about 60 seconds to record) indicated that it was helpful in at least one of the following ways: (1) reducing the severity or duration of the PA, (2) learning about their personalized fear response, or (3) feeling more in control of their body. The participants who used the app rated the user difficulty as 4.73 (SD 2.57) out of 10.

Table 2 presents a qualitative analysis of participant comments from the final survey (n=16). In response to an open-ended question, “Overall, in what ways did you find app use helpful?” the most frequent responses were about observing symptom patterns, which aided understanding of body response (symptom pattern recognition) and the structure of the app helping to redirect user attention (guided attention). In response to the open-ended question, “Overall, what challenges did you face in using the app during panic attacks?” the most frequent responses were about remembering or being motivated enough to open the app (forgot or unmotivated to use) while experiencing panic and app problems owing to technology glitch or user error (such as difficulty placing finger properly for HR to be detected) being a barrier to use (technology difficulties). Representative testimonials and additional response categories are presented in Table 2. When asked if they would use the app again in spite of difficulties, 56% (9/16) of the participants indicated they would. When asked if they would recommend the app to someone else experiencing panic, 94% (15/16) of the participants indicated they would recommend it. In examining the discrepancy between the prevalence of future use and recommendation to others, it appeared that some participants believed the app could be helpful to others who may better remember to open the app at the start of panic symptoms.

**Table 1:** Pilot study participant demographics (N=18).

Pilot study demographics					Values
**Gender, n (%)**					
	Female			13 (72)	
	Male			3 (17)	
	Other			2 (11)	
Age (years), mean (SD)^a^					24 (5)
**Race, n (%)**					
	White			15 (83)	
	Asian American			1 (6)	
	Other			2 (11)	
**Ethnicity, n (%)**					
	Non-Hispanic			16 (89)	
	Hispanic			2 (11)	
**Mental health diagnosis, n (%)**					
	Any diagnosis			13 (72)	
	**Type of diagnosis**				
		Anxiety	10 (56)		
		Depression	7 (39)		
		OCD^b^	3 (17)		
		PTSD^c^	3 (17)		
		ADHD^d^	2 (11)		
		Panic	2 (11)		
		Eating disorder	2 (11)		
		Bipolar disorder	1 (6)		
		Adjustment disorder	1 (6)		
		Personality disorder	1 (6)		
**Panic treatment sought, n (%)**					
	Currently in therapy			12 (67)	
	Ever in therapy			17 (94)	
	Physician			13 (72)	
	Emergency room or urgent care			5 (28)	
	Prescribed medication			10 (56)	
	Self-medicated (alcohol or drugs)			5 (28)	
	Biofeedback			0 (0)	

^a^Range: 19-35 years.

^b^OCD: obsessive compulsive disorder.

^c^PTSD: posttraumatic stress disorder.

^d^ADHD: attention-deficit/hyperactivity disorder.

**Table 2:** Pilot study content analysis (n=16).

Qualitative category		Values, n (%)	Example testimonial 1	Example testimonial 2
**Overall, in what ways did you find app use helpful?**				
	Symptom pattern recognition (observing symptom patterns aided the understanding of body response)	6 (38)	“Rather than freaking out and feeling like I was dying, I saw that my heart rate was just slightly elevated and fluctuating, and I knew that the attack was temporary and I could work through it.”	“I liked how it kept track of my heart rate because seeing it decrease was calming.”
	Guided attention (the structure redirected attention)	6 (38)	“Watching my heart rate during the panic attack helped me focus more on what was going on.”	“Seeing my pulse change was really helpful in giving me something to focus on to calm down during a panic attack.”
	Accessibility (knowing it was accessible)	3 (19)	“The app provides instant assistance with attacks, instead of waiting to get help.”	“The app was accessible from my pocket, easy to read, and easy to follow.”
	Physiological validation (the personalized data objectively acknowledged the experience as a panic attack)	3 (19)	“The app was most helpful in getting me to acknowledge experiences that I've had for a long time as real, manageable symptoms of a known disorder - rather than just terrifying feelings.”	“I found the information provided by the app regarding panic attacks to be calming and affirming.”
	Affirmations (the words of encouragement)	2 (13)	“The positive affirmations it gives you is helpful.”	“It helped encourage me through it and stay in tune with myself.”
	Triggers (being asked to identify triggers)	2 (13)	“Identifying the trigger and watching your body calm down as you calm down was helpful.”	“The app has many different triggers that we could choose from.”
**Overall, what challenges did you face in using the app during panic attacks?**				
	Forgot or unmotivated to use (remembering or being motivated to open the app owing to panic)	8 (50)	“I often lacked the presence of mind or motivation to get my phone and start tracking it.”	“It is not my first instinct to use an app when I am having an attack.”
	Technology difficulties (owing to glitch or user error)	5 (31)	“Sometimes I felt like it wasn't recording my pulse right which I fixated on.”	“The app never gave me an average length of my panic attacks so it always said I had 0 min left.”
	Symptom barrier (panic symptoms impacted app use once it was opened)	4 (25)	“Physically shaking made it hard for me to keep my finger on long enough to read my heart rate.”	“I had trouble answering because I was freaking out.”
	Not accessible (did not have access to phone)	3 (19)	“I didn't end up having my phone with me during most of my panic attacks.”	“It's too inconvenient for me to use considering my panic attacks often happen while driving.”
	Repetitive guidance (structure of app was repetitive)	1 (6)	“Questions too repetitive, especially when tracking more than one attack a day.”	—^a^
**Would you use the app again?**				
	Yes	9 (57)	“To monitor myself and keep myself in touch with my body and reality.”	“It was helpful so I would continue using it.”
**Would you recommend the app?**				
	Yes	15 (94)	“I would say yes because it made me feel more educated on my physical well-being.”	“Although it doesn't work for me [Forgot to Use], I definitely recognize the benefit of real time biofeedback, and I feel like this is a great option for people who struggle with anxiety and panic attacks.”

^a^Second testimonial is not available.

## Discussion

### Summary

#### Overview

In this study, we examined the profile of real-world PAs recorded by users of the mHealth app, PanicMechanic, in the largest observation of PA physiology yet considered in this research area (N=50). We also assessed the feasibility and usefulness of the app as a PA intervention in a pilot study of university students (N=18). We discuss the results of these studies in the following sections, including an analysis of the characteristic changes in HR and anxiety ratings during PAs, common lifestyle contributors and triggers for PAs, and the observations of the pilot study participants after using PanicMechanic for the 12-week intervention period.

#### Profiles of Real-world PAs

During recorded PAs, HR appears to fluctuate by approximately 15 bpm (Figure 3), which is consistent with several studies that reported significant HR change at the onset of a PA [6]. However, other studies with null results identified a HR increase only when HR was *disproportionate to activity levels* [8]. Our results show that the average HR varied between 85 and 100 bpm, which are within normal limits of resting HR for adults [34] and therefore would not qualify as HR increase under that study criterion. It may be noted that although there is a significant difference between PA HR peak and baseline, it is the *relative* HR increase that indicates a PA. For reference, an increase of approximately 15 bpm is seen in 30 seconds of stair climbing in a healthy individual aged 44 years [35].

On the basis of data from the PanicMechanic users, there appears to be an average duration from peak to baseline HR of approximately 30 seconds during PAs (Figure 3). Given that previous works indicate that PAs last approximately 10 minutes, these results imply that there are several cycles of HR peaks and recovery to baseline within a PA. Although the frequency of HR changes during PAs has not been reported previously, a study appears to show that 4 HR cycles occur within a typical 10-minute PA (Figure 2) [6]. Similarly, the data we report in Figure 2 show 3 clear HR cycles during a 5-minute PA recording. This cyclical HR pattern could be beneficial to biofeedback intervention, allowing multiple chances for patients to observe and expect recovery patterns. From the data presented in Figure 2, we note that significant heterogeneity was observed in the pattern of HR during the recorded PAs across users. Further exploration of this heterogeneity remains an interesting potential area of future study as these HR patterns could further inform our understanding of how and when PAs induce cycles of physiological arousal.

Subjective anxiety did not follow a cyclical pattern, but slowly decreased across the HR monitoring period (Figure 3). This is consistent with research demonstrating that physiological data does not necessarily correlate with subjective anxiety ratings during fear tasks (eg, tasked with giving a spontaneous speech in front of judges [36]). In previous literature, a significant HR increase was seen to precede self-reported PA onset by approximately 1 minute [6]; thus, there may be a sequential nonlinear relationship between HR and subjective anxiety outside the scope of our recorded PA profile. Observing moment-by-moment HR metrics alongside self-reported anxiety may allow users, or, importantly, help clinicians to observe and expect panic patterns that they may not be able to intervene on otherwise.

We also derived profiles of PA triggers (of the available choices), which were not previously examined. As expected, because this mHealth app became available during the COVID-19 pandemic in the United States (released in April 2020), health was the primary trigger in more than one-third of PAs (Figure 4). This is supported by the pilot study data in which the participants indicated that the pandemic increased the frequency, severity, and duration of their PAs. On days where PAs were recorded, the users reported slightly worse stress, sleep, and eating habits; slightly less exercise than typical; and slightly less drug or alcohol consumption in the past 24 hours (Figure 4). These results could inform future studies focused on predictive models of PA occurrence by providing objective monitoring of these parameters or behavioral interventions to reduce PA risk.

#### PanicMechanic as a PA Intervention

The pilot study results provide insight into the populations that are most interested in participating in an mHealth intervention for PAs. Results show that most participants had multiple mental health disorders (Table 1), consistent with previous literature on individuals who experience PAs [37]. Of the 18 participants, 2 (11%) participants had PD (observed in 3.8% of persons with PAs in the general public [1]). In addition, consistent with previous literature, the participants (all experienced a PA in the month before study participation) had also sought several forms of help services before the study [2] but, importantly, had never tried biofeedback. Interestingly, all the participants reported experiencing subjective HR increases during PAs (Figure 5), suggesting the validity of HR-based biofeedback.

We assessed the subjective feasibility of using PanicMechanic as a biofeedback intervention for PAs. Quantitatively, the participants rated the difficulty of use as a 4.73 out of 10. Qualitatively, half of the participants indicated that the main difficulty of app use was not remembering or being motivated to open the app during the onset of a PA. Despite difficulties, 56% (9/16) of the participants indicated that they would use the app again in the future. Future development work to incorporate hearables and wrist-worn or other wearable devices is indicated to capture HR continuously and more seamlessly. We envision a system in which a push notification asks the user to open the PanicMechanic app if a HR increase has occurred outside of exercise, which may precede a PA [6]. This type of system modification could, for example, reduce the challenge of not remembering to open the app at the beginning of a PA.

However, despite these challenges, 94% (15/16) of the participants would recommend the PanicMechanic app to others who have PAs. Interestingly, several participants indicated that they would not use the app themselves but would recommend it. These participants cited that they believed in the theory of the intervention, but that their use challenges such as forgetting to use the app or their own circumstances such as only experiencing PAs while driving prevented them from benefitting from the intervention. Quantitatively and qualitatively, the participants found this biofeedback app useful. In weekly reports, 90% (14/16) of the participants who had valid use of the app (at least 4 HR data points, which would take at least 60 seconds to record) indicated that PanicMechanic was helpful in one of the following three ways: (1) reducing the severity or duration of the PA, (2) learning about their personalized fear response, or (3) feeling more in control of their body. Qualitatively, they reported that observing their personalized symptom patterns aided their own understanding of their body and also that the app helped them focus by guiding them through their panic in a structured way. These mechanisms are consistent with previous theory on how biofeedback works [17] and indicate that PanicMechanic shows promise as an accessible PA intervention.

There are several limitations to our study. First, we cannot be certain of the validity and duration of the recorded PAs. It is possible that users recorded HR data outside of a PA, started to record data after PA onset, and completed data recording before the end of a PA. We attempted to mitigate these problems by providing a step-by-step tutorial and *practice* mode on the app upon download and only analyzing PAs that had at least 4 HR measurements with a visible peak. Importantly, these steps yielded exclusion of 66% of our HR data. Thus, although collection of unsupervised data allows for much larger sample sizes than previously studied, the available data diminishes as it is cleaned [6-9]. As the app provided biofeedback during the first PA recording, it is possible that PAs may be attenuated in intensity owing to biofeedback, as the effective number of sessions studied ranged from 1 to 50 [26]. In addition, we report the prevalence of triggers of remote PAs. However, it is important to note that in this iteration of PanicMechanic, there were specific choices of triggers and no ability to input other options. Users were also able to track a PA without indicating a trigger. These prelisted triggers likely do not encompass all the triggers of PAs and thus the results can only represent the prevalence of the given triggers. Given the likely need for multiple sessions for PA improvement and the fact that only 5 (31%) of the 16 participants in our pilot study used the app more than twice, we were not able to evaluate the objective effectiveness of PanicMechanic. This should be analyzed in future studies of app users and specifically in a controlled trial. Overall, our pilot study sample size was small and they represented a subsample of all the people who have PAs. Future studies should include a larger sample with a more diverse range of people who have PAs to inform more generalizable conclusions.

### Conclusions

Overall, we have presented a small window into the profile of real-world PAs in the largest sample of people who have PAs to date. We also demonstrated promising preliminary results from a pilot study with participants indicating that PanicMechanic is useful, albeit with some feasibility challenges, which can be addressed with simple app improvements that leverage existing technologies.

## Abbreviations

bpm: beats per minute
BT: breathing training
HR: heart rate
mHealth: mobile health
PA: panic attack
PD: panic disorder
